# Crystal structure of a 1,1-dibutyl-1*H*,3*H*-naphtho[1,8-*cd*][1,2,6]oxastannaborinin-3-ol

**DOI:** 10.1107/S2056989021000712

**Published:** 2021-01-26

**Authors:** Kevin Breitwieser, Peter Chen

**Affiliations:** a Laboratorium für Organische Chemie, ETH Zürich, Zürich, Switzerland

**Keywords:** crystal structure, boron, tin, heterocycle, stannaborininol

## Abstract

The title oxastannaborininol compound, [Sn(C_4_H_9_)_2_(C_10_H_7_BO_2_)], has been synthesized and crystallized. While heterocycles containing a C–O–B group are common, heterocycles containing an *E*–O–B unit, where *E* is an element of the carbon group except for carbon, are rare. In fact, while heterocycles containing Si–O–B units are occasionally reported (although without crystal structures), there are no reports for the corresponding germanium, tin or lead analogues. Herein, the first synthesis and crystal structure of a heterocycle containing an Sn–O–B unit is described. The asymmetric unit contains one mol­ecule showing a notable disorder of the tin atom and the butyl groups. They occupy two sets of positions with site-occupancy factors of 0.295 (6) and 0.705 (6).

## Abstract 2

A novel oxastannaborininol, 1,1-dibutyl-1*H*,3*H*-naphtho­[1,8-*cd*][1,2,6]oxastannaborinin-3-ol, has been synthesized and crystallized. It is the first reported compound with a heterocycle containing an Sn–O–B unit.

## Chemical context   

Both tin and boron organic compounds are widespread reagents for cross-coupling reactions in organic synthesis (Negishi, 2002 ▸). The combination of tin- and boron-containing groups in one mol­ecule can be advantageous, as they can undergo cross-coupling under different conditions. While the stannyl group easily undergoes transmetalation at elevated temperatures, a boronic acid will not do so with an additional activator, usually a base (Cárdenas, 2003 ▸). However, those groups are not usually connected. The only reported use of esters of stannanols and boronic acids lies in their increased Lewis acidity compared to the free boronic acid (Beckett *et al.*, 1999 ▸). They have been otherwise mentioned only in one publication, although no applications were reported (Murphy *et al.*, 1993 ▸).

**Figure 1:**
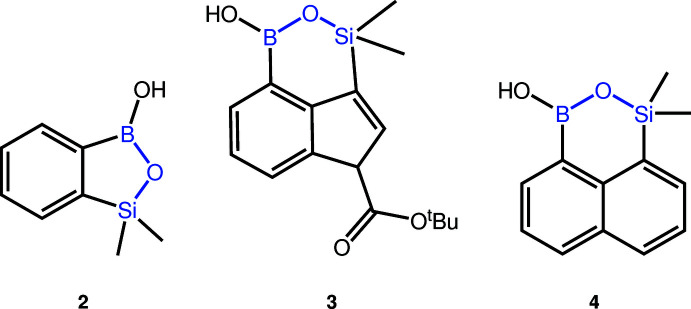
Chemical structure of compounds known in the literature that contain a heterocycle with an Si–O–B unit. Compound **2** (Brzozowska *et al.*, 2015 ▸) is a benzosiloxaborole, while compound **3** (Sumida *et al.*, 2018 ▸) and compound **4** (Su *et al.*, 2018 ▸) are oxasilaborininols.

Heterocycles containing an *E*–O–B unit (*E* = Si, Ge, Sn, Pb) have so far only been reported for silicon (Fig. 1 ▸). Benzosiloxaboroles, containing a five-membered ring with an Si–O–B unit, have shown promising properties for medical applications, being strong anti­microbial (Durka *et al.*, 2019 ▸) and anti­fungal agents (Brzozowska *et al.*, 2015 ▸).

Oxasilaboroninols have only been described in two cases. Sumida and co-workers accidentally stumbled upon **3** while trying to synthesize an oxasilole. They showed that both organometallic moieties can be replaced successively through Suzuki–Miyaura and Hiyama coupling (Sumida *et al.*, 2018 ▸). Su and Hartwig on the other hand synthesized oxasiliaboroninol **4** using ruthenium catalysis (Su *et al.*, 2018 ▸). In their report, they describe multiple transformations for this product, being able to replace selectively the boronic acid group while leaving a silanol group behind.
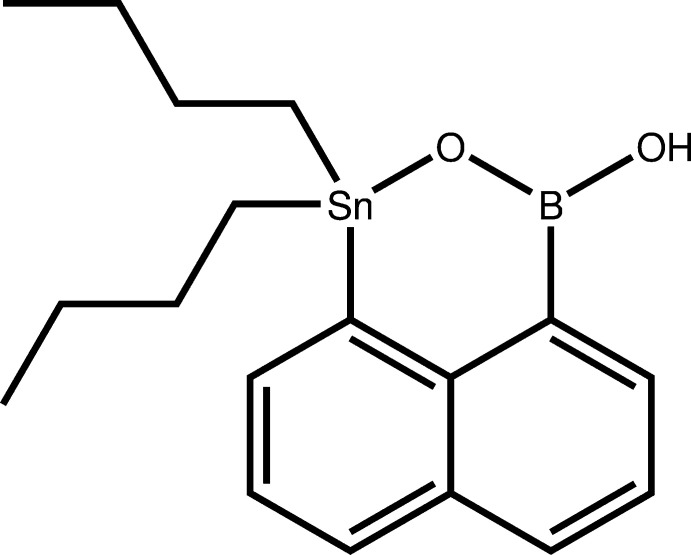


## Structural commentary   

**Figure 2:**
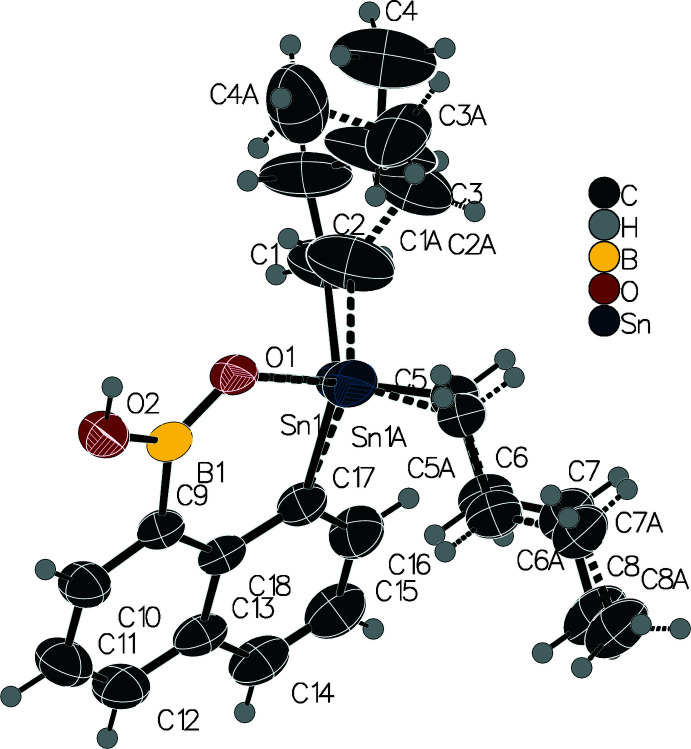
Crystal structure of the title compound **1**. Displacement ellipsoids are drawn at the 50% probability level. Hydrogen atoms are drawn as fixed-size spheres with a radius of 0.15 Å. The tin atom and the butyl groups show notable disorder.

The title mol­ecule (**1**) is a cyclic intra­molecular ester of a boronic acid and a stannanol. The asymmetric unit contains one mol­ecule (Fig. 2 ▸). It shows notable disorder of the tin atom and the butyl groups. They occupy two sets of positions with site-occupancy factors of 0.295 (6) and 0.705 (6). It is furthermore planar, pointing towards electron delocalization over almost the whole mol­ecule. The C—C bond lengths in the naphthalene structure are between 1.352 (4) and 1.439 (3) Å. This is in line with the bond lengths in naphthalene ranging from 1.350 to 1.421 Å (Abrahams *et al.*, 1949 ▸). The Sn—O bond distance is 2.0041 (17) and 2.040 (3) Å and the Sn—C bond connecting the tin atom to the aromatic ring has a length of 2.151 (6) Å and 2.210 (4) Å, varying due to disorder. The B—C bond has a length of 1.594 (3) Å, the B—O bond lengths are 1.352 (3) Å (B—OSn) and 1.362 (3) Å (B—OH).

## Supra­molecular features   

**Figure 3:**
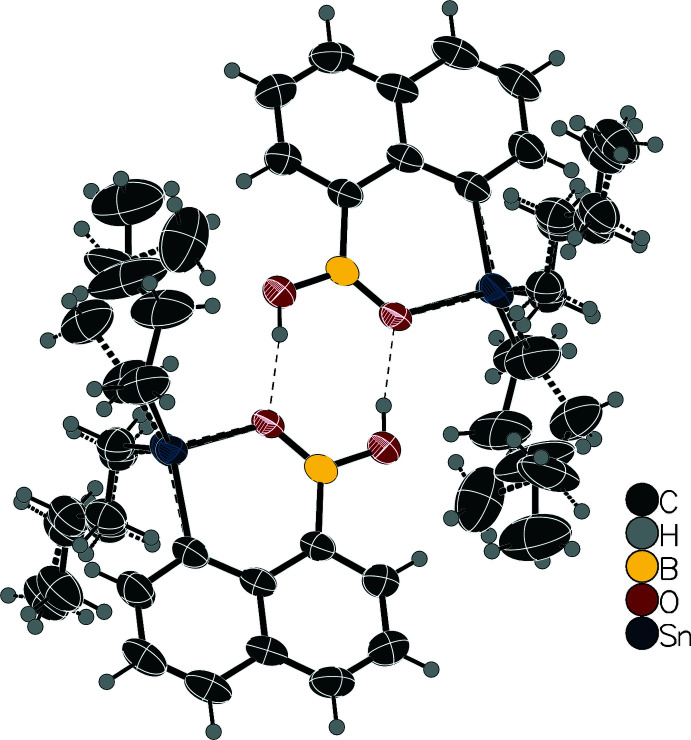
Structure of the dimer formed through hydrogen bonding. Displacement ellipsoids are drawn at the 50% probability level. Hydrogen atoms are drawn as fixed-size spheres with a radius of 0.15 Å.

In the crystal, the mol­ecules form dimers through pairs of hydrogen bonds between the ring oxygen atom and the hydroxyl group with a distance of 2.805 (2) Å between the two involved oxygen atoms (Fig. 3 ▸, Table 1 ▸).

## Database survey   

**Figure 4:**
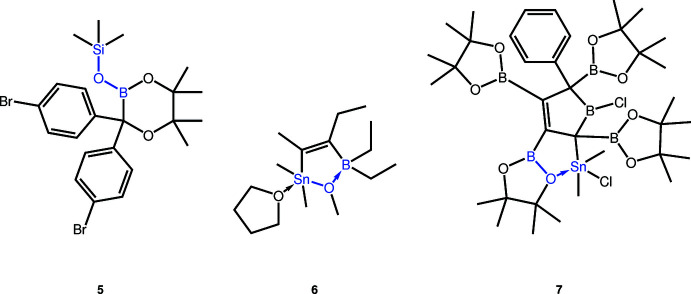
Structures of the compounds with the C–B–O–*E*–C motif (*E* = Si, Ge, Sn, Pb) and reported crystal structures. Compound **5** is the only one where this motif is formed by covalent bonds only (Ito *et al.*, 2011 ▸), while the compounds **6** (Boese *et al.*, 1996 ▸) and **7** (Braunschweig *et al.*, 2017 ▸) contain coordinative bonds.

Searching the Cambridge Structural Database (CSD, version 5.41, update of November 2019; Groom *et al.*, 2016 ▸), not a single ester of a boronic acid and a stannanol has been crystallized. The same is true for the corresponding germanol and plumbanol derivatives. One ester of a boronic acid and tri­methyl­silanol (**5**) has been crystallized (Ito *et al.*, 2011 ▸). Two additional crystal structures containing the C–B–O–Sn–C motif have been reported. However, in those cases, either the O—Sn bond in **6** (Braunschweig *et al.*, 2017 ▸) or the O—B bond in **7** (Boese *et al.*, 1996 ▸) are not covalent, but rather coordinative bonds. Those three mol­ecules are shown in Fig. 4 ▸.

The CSD lists three stannanols, all of which are triaryl stannanols (Růžička *et al.*, 2013 ▸; Barbul *et al.*, 2012 ▸). For those compounds, the Sn—O bond has a length of 1.981 to 2.057 Å, agreeing with the bond length of 2.0041 (17) Å found for the title compound. The Sn—C_Ar_ bond length varies between 2.143 and 2.208 Å, matching the corresponding bond in the title compound.

## Synthesis and crystallization   

8-Iodo-1-naphthyl­boronic acid was prepared according to literature (Katz, 1986 ▸). Under argon, 122.6 mg (0.412 mmol, 1 eq.) of 8-iodo-1-naphthyl­boronic acid and 0.13 mL (0.459 mmol, 1.1 eq.) of tri­butyl­tin methoxide were heated to 373 K for 22.5 h; 0.2 mL (0.706 mmol, 1.7 eq.) of tri­butyl­tin methoxide were added and stirring was continued for 21 h at 373 K. Then 0.5 mL (1.764 mmol, 4.3 eq.) of tri­butyl­tin methoxide were added and the mixture was heated to 403 K for an additional 23 h. The mixture was cooled to RT and diluted by the addition of hexane. It was washed with equal volume 1 *M* aq. NaOH, dried (Na_2_SO_4_), filtered and concentrated *in vacuo*. The residue was purified by column chromatography (pure hexane to hexa­ne:ethyl acetate 1:1) to obtain a yellowish solid that was crystallized by slow evaporation of a solution in 1,2-di­meth­oxy­ethane at 258 K and washed with pentane to obtain 27.3 mg (0.068 mmol, 15%) of colorless crystals suitable for X-ray crystallography.

^1^H NMR (400 MHz, CDCl_3_) δ 8.44 (*dd*, *J* = 7.0, 1.5 Hz, 1H, H12), 7.93 (*dd*, *J* = 8.2, 1.5 Hz, 1H, H10), 7.89 (*dd*, *J* = 7.1, 2.6 Hz, 1H, H14), 7.55 (*dd*, *J* = 8.1, 7.0 Hz, 1H, H11), 7.51–7.38 (*m*, 2H, H15 & H16), 4.80–4.37 (*s*, 1H, OH), 1.67 (*dtd*, *J* = 14.3, 7.2, 2.5 Hz, 4H, H2*A* & H2*B* & H6*A* & H6*B*), 1.47–1.29 (*m*, 8H H1*A* & H1*B* & H3*A* & H3*B* & H5*A* & H5*B* & H7*A* & H7*B*), 0.87 (*t*, *J* = 7.3 Hz, 6H H4*A* & H4*B* & H4*C* & H8*A* & H8*B* & H8*C*).

^11^B NMR (128 MHz, CDCl_3_) δ 27.22 (*s*, *br*, B1).

^13^C NMR (101 MHz, CDCl_3_) δ 142.65 (*s*, C18), 139.20 (*s*, C13), 137.30 (*s*, C12), 134.50 (*s*, C16), 133.78 (*s*, C17), 132.13 (*s*, C10), 130.81 (*s*, C14), 125.86 (*s*, C11), 124.55 (*s*, C15), 27.55 (*s*, C2 & C6), 27.10 (*s*, C3 & C7), 17.51 (*s*, C1 & C5), 13.70 (C4 & C8). C9 is not visible due to C–B inter­actions.

## Refinement   

Crystal data, data collection and structure refinement details are summarized in Table 2 ▸. Data were collected at 200 K, as a phase transition leads to breaking crystals at lower temperatures. The disordered tin atom and butyl groups were restrained using rigid body (RIGU) restraints with σ for 1–3 distances and 1–2 distances of 0.004 and same-distance (SADI) restrains were applied to equivalent 1,2- and 1,3-distances within the disorder. Ellipsoids of four atoms and their equivalents in the alternate orientation were constrained to be equal (EADP). H atoms were refined with riding coordinates [C—H = 0.93–0.97; *U*_iso_(H) = 1.2*U*_eq_(C) or 1.5*U*_eq_(O, C-meth­yl)] except for the proton involved in the hydrogen bond, which was only lightly restrained with DFIX.

## Supplementary Material

Crystal structure: contains datablock(s) I. DOI: 10.1107/S2056989021000712/zq2260sup1.cif

Structure factors: contains datablock(s) I. DOI: 10.1107/S2056989021000712/zq2260Isup2.hkl

CCDC reference: 2057745

Additional supporting information:  crystallographic information; 3D view; checkCIF report

## Figures and Tables

**Table 1:** Hydrogen-bond geometry (Å, °)

*D*—H⋯*A*	*D*—H	H⋯*A*	*D*⋯*A*	*D*—H⋯*A*
O2—H2⋯O1^i^	0.88 (2)	1.93 (2)	2.805 (2)	172 (3)

Symmetry code: (i) 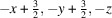.

**Table 2:** Experimental details

Crystal data	
Chemical formula	[Sn(C_4_H_9_)_2_(C_10_H_7_BO_2_)]
*M*_r_	402.88
Crystal system, space group	Monoclinic, *C*2/*c*
Temperature (K)	200
*a*, *b*, *c* (Å)	30.1386 (6), 11.2948 (1), 16.4726 (3)
β (°)	139.457 (4)
*V* (Å^3^)	3644.9 (2)
*Z*	8
Radiation type	Cu *K*α
μ (mm^−1^)	11.17
Crystal size (mm)	0.26 × 0.13 × 0.02

Data collection	
Diffractometer	Rigaku Oxford Diffraction XtaLAB Synergy, Dualflex, Pilatus 300K
Absorption correction	Gaussian (*CrysAlis PRO*; Rigaku OD, 2018 ▸)
*T*_min_, *T*_max_	0.234, 1.000
No. of measured, independent and observed [*I* > 2σ(*I*)] reflections	28847, 3923, 3724
*R*_int_	0.033
(sin θ/λ)_max_ (Å^−1^)	0.638

Refinement	
*R*[*F* ^2^ > 2σ(*F* ^2^)], *wR*(*F* ^2^), *S*	0.024, 0.066, 1.08
No. of reflections	3923
No. of parameters	264
No. of restraints	167
H-atom treatment	H atoms treated by a mixture of independent and constrained refinement
Δρ_max_, Δρ_min_ (e Å^−3^)	0.45, −0.39

Computer programs: *CrysAlis PRO* (Rigaku OD, 2018 ▸), *SHELXT* (Sheldrick, 2015*a* ▸), *SHELXL2018/3* (Sheldrick, 2015*b* ▸) and *OLEX2* (Dolomanov *et al.*, 2009 ▸).

## supplementary crystallographic information

### Crystal data

**Table:**

[Sn(C_4_H_9_)_2_(C_10_H_7_BO_2_)]	*F*(000) = 1632
*M**_r_* = 402.88	*D*_x_ = 1.468 Mg m^−^^3^
Monoclinic, *C*2/*c*	Cu *K*α radiation, λ = 1.54184 Å
*a* = 30.1386 (6) Å	Cell parameters from 21527 reflections
*b* = 11.2948 (1) Å	θ = 4.5–79.4°
*c* = 16.4726 (3) Å	µ = 11.17 mm^−^^1^
β = 139.457 (4)°	*T* = 200 K
*V* = 3644.9 (2) Å^3^	Plate, clear colourless
*Z* = 8	0.26 × 0.13 × 0.02 mm

### Data collection

**Table:**

Rigaku Oxford Diffraction XtaLAB Synergy, Dualflex, Pilatus 300K diffractometer	3724 reflections with *I* > 2σ(*I*)
ω scans	*R*_int_ = 0.033
Absorption correction: gaussian (CrysAlisPro; Rigaku OD, 2018)	θ_max_ = 79.6°, θ_min_ = 4.5°
*T*_min_ = 0.234, *T*_max_ = 1.000	*h* = −38→29
28847 measured reflections	*k* = −14→14
3923 independent reflections	*l* = −17→20

### Refinement

**Table:**

Refinement on *F*^2^	Primary atom site location: dual
Least-squares matrix: full	Hydrogen site location: mixed
*R*[*F*^2^ > 2σ(*F*^2^)] = 0.024	H atoms treated by a mixture of independent and constrained refinement
*wR*(*F*^2^) = 0.066	*w* = 1/[σ^2^(*F*_o_^2^) + (0.0359*P*)^2^ + 2.7329*P*] where *P* = (*F*_o_^2^ + 2*F*_c_^2^)/3
*S* = 1.08	(Δ/σ)_max_ = 0.001
3923 reflections	Δρ_max_ = 0.45 e Å^−^^3^
264 parameters	Δρ_min_ = −0.39 e Å^−^^3^
167 restraints	

### Special details

**Table:**

Geometry. All esds (except the esd in the dihedral angle between two l.s. planes) are estimated using the full covariance matrix. The cell esds are taken into account individually in the estimation of esds in distances, angles and torsion angles; correlations between esds in cell parameters are only used when they are defined by crystal symmetry. An approximate (isotropic) treatment of cell esds is used for estimating esds involving l.s. planes.

### Fractional atomic coordinates and isotropic or equivalent isotropic displacement parameters (Å^2^)

**Table:**

	*x*	*y*	*z*	*U*_iso_*/*U*_eq_	Occ. (<1)
Sn1	0.80127 (4)	0.57065 (8)	0.24979 (9)	0.03908 (16)	0.705 (6)
O1	0.75421 (8)	0.66412 (13)	0.09740 (13)	0.0452 (3)	
O2	0.66330 (9)	0.77545 (16)	−0.07920 (16)	0.0560 (4)	
H2	0.6923 (15)	0.790 (3)	−0.079 (3)	0.084*	
C18	0.65789 (11)	0.61897 (17)	0.12184 (19)	0.0411 (4)	
C17	0.72252 (13)	0.56497 (18)	0.2265 (2)	0.0465 (5)	
C9	0.64190 (11)	0.68493 (18)	0.02737 (19)	0.0427 (4)	
C13	0.60618 (13)	0.6048 (2)	0.1109 (2)	0.0498 (5)	
C16	0.73393 (15)	0.5022 (2)	0.3129 (3)	0.0606 (6)	
H16	0.776391	0.467551	0.380439	0.073*	
C12	0.54116 (13)	0.6536 (3)	0.0087 (3)	0.0628 (6)	
H12	0.507856	0.643613	0.002056	0.075*	
C15	0.68324 (17)	0.4895 (3)	0.3016 (3)	0.0676 (7)	
H15	0.692174	0.447318	0.361363	0.081*	
B1	0.69046 (12)	0.7075 (2)	0.0188 (2)	0.0403 (4)	
C14	0.62094 (16)	0.5390 (2)	0.2029 (3)	0.0609 (6)	
H14	0.587231	0.529701	0.195310	0.073*	
C10	0.57682 (13)	0.7313 (2)	−0.0696 (2)	0.0571 (6)	
H10	0.565960	0.775310	−0.130655	0.069*	
C11	0.52614 (14)	0.7150 (3)	−0.0803 (3)	0.0698 (7)	
H11	0.482665	0.746192	−0.148228	0.084*	
C7	0.9197 (5)	0.8346 (9)	0.5547 (9)	0.0641 (11)	0.705 (6)
H7A	0.927199	0.892715	0.522732	0.077*	0.705 (6)
H7B	0.962017	0.791753	0.621729	0.077*	0.705 (6)
C6	0.8653 (4)	0.7496 (5)	0.4523 (7)	0.0553 (11)	0.705 (6)
H6A	0.823204	0.793127	0.385509	0.066*	0.705 (6)
H6B	0.857484	0.692717	0.484475	0.066*	0.705 (6)
C1	0.8221 (5)	0.3983 (7)	0.2290 (11)	0.0757 (19)	0.705 (6)
H1A	0.854211	0.357484	0.308673	0.091*	0.705 (6)
H1B	0.780202	0.352665	0.169737	0.091*	0.705 (6)
C5	0.8825 (3)	0.6821 (5)	0.3978 (6)	0.0555 (11)	0.705 (6)
H5A	0.923203	0.634959	0.462947	0.067*	0.705 (6)
H5B	0.891816	0.738315	0.367705	0.067*	0.705 (6)
C8	0.9005 (7)	0.8980 (10)	0.6062 (8)	0.0776 (15)	0.705 (6)
H8A	0.855991	0.932701	0.538591	0.116*	0.705 (6)
H8B	0.933280	0.959032	0.662845	0.116*	0.705 (6)
H8C	0.900010	0.842311	0.649544	0.116*	0.705 (6)
C3A	0.9362 (9)	0.469 (2)	0.2939 (13)	0.131 (9)	0.295 (6)
H3AA	0.980912	0.440122	0.340706	0.158*	0.295 (6)
H3AB	0.940412	0.552561	0.313166	0.158*	0.295 (6)
C2	0.8516 (4)	0.4040 (4)	0.1841 (8)	0.110 (2)	0.705 (6)
H2A	0.859231	0.324071	0.175231	0.132*	0.705 (6)
H2B	0.818392	0.441013	0.102315	0.132*	0.705 (6)
C4	0.9615 (5)	0.4669 (7)	0.2585 (10)	0.142 (3)	0.705 (6)
H4A	0.943675	0.519424	0.193075	0.213*	0.705 (6)
H4B	0.962191	0.387520	0.238623	0.213*	0.705 (6)
H4C	1.007051	0.490615	0.335834	0.213*	0.705 (6)
C4A	0.8861 (8)	0.4541 (14)	0.1525 (11)	0.117 (6)	0.295 (6)
H4AA	0.840486	0.445560	0.109470	0.176*	0.295 (6)
H4AB	0.898299	0.384852	0.138891	0.176*	0.295 (6)
H4AC	0.888484	0.522479	0.121278	0.176*	0.295 (6)
C3	0.9174 (5)	0.4718 (7)	0.2707 (12)	0.149 (5)	0.705 (6)
H3A	0.906412	0.554416	0.264650	0.179*	0.705 (6)
H3B	0.945236	0.446432	0.354910	0.179*	0.705 (6)
C2A	0.9121 (6)	0.4051 (10)	0.3302 (14)	0.091 (4)	0.295 (6)
H2AA	0.941594	0.426381	0.416263	0.110*	0.295 (6)
H2AB	0.919817	0.321779	0.330202	0.110*	0.295 (6)
C1A	0.8408 (11)	0.4164 (14)	0.261 (3)	0.100 (8)	0.295 (6)
H1AA	0.833837	0.357857	0.293132	0.120*	0.295 (6)
H1AB	0.810547	0.396319	0.173984	0.120*	0.295 (6)
Sn1A	0.8101 (2)	0.5864 (4)	0.2649 (3)	0.0696 (7)	0.295 (6)
C5A	0.8830 (9)	0.7164 (16)	0.4067 (17)	0.0555 (11)	0.295 (6)
H5AA	0.926737	0.677223	0.474024	0.067*	0.295 (6)
H5AB	0.888251	0.774727	0.371250	0.067*	0.295 (6)
C6A	0.8671 (11)	0.7814 (15)	0.4627 (19)	0.0553 (11)	0.295 (6)
H6AA	0.827565	0.831359	0.398553	0.066*	0.295 (6)
H6AB	0.854627	0.723469	0.486213	0.066*	0.295 (6)
C7A	0.9231 (13)	0.856 (3)	0.574 (2)	0.0641 (11)	0.295 (6)
H7AA	0.931733	0.920516	0.547875	0.077*	0.295 (6)
H7AB	0.964334	0.808857	0.633969	0.077*	0.295 (6)
C8A	0.9093 (18)	0.908 (3)	0.637 (2)	0.0776 (15)	0.295 (6)
H8AA	0.879434	0.974810	0.589615	0.116*	0.295 (6)
H8AB	0.951534	0.932796	0.719433	0.116*	0.295 (6)
H8AC	0.888151	0.849241	0.640933	0.116*	0.295 (6)

### Atomic displacement parameters (Å^2^)

**Table:**

	*U*^11^	*U*^22^	*U*^33^	*U*^12^	*U*^13^	*U*^23^
Sn1	0.0426 (2)	0.0423 (2)	0.04336 (19)	0.00587 (15)	0.03572 (17)	0.00775 (15)
O1	0.0546 (8)	0.0530 (8)	0.0488 (7)	0.0110 (6)	0.0450 (7)	0.0129 (6)
O2	0.0562 (9)	0.0733 (11)	0.0550 (9)	0.0151 (8)	0.0468 (8)	0.0221 (8)
C18	0.0557 (11)	0.0377 (9)	0.0490 (10)	−0.0046 (8)	0.0451 (10)	−0.0059 (8)
C17	0.0602 (13)	0.0475 (11)	0.0514 (12)	−0.0012 (9)	0.0478 (11)	0.0012 (8)
C9	0.0521 (11)	0.0445 (10)	0.0475 (10)	0.0001 (8)	0.0423 (10)	−0.0010 (8)
C13	0.0656 (13)	0.0488 (11)	0.0632 (13)	−0.0119 (10)	0.0568 (12)	−0.0116 (10)
C16	0.0771 (16)	0.0629 (15)	0.0624 (14)	0.0033 (12)	0.0587 (14)	0.0109 (11)
C12	0.0598 (14)	0.0779 (17)	0.0756 (16)	−0.0075 (12)	0.0584 (14)	−0.0072 (13)
C15	0.095 (2)	0.0702 (16)	0.0759 (17)	−0.0076 (15)	0.0759 (17)	0.0055 (13)
B1	0.0510 (12)	0.0406 (10)	0.0437 (11)	0.0022 (9)	0.0399 (10)	0.0014 (9)
C14	0.0834 (17)	0.0627 (14)	0.0767 (16)	−0.0206 (13)	0.0720 (16)	−0.0132 (13)
C10	0.0570 (13)	0.0690 (15)	0.0602 (13)	0.0105 (11)	0.0486 (12)	0.0125 (11)
C11	0.0553 (14)	0.091 (2)	0.0741 (17)	0.0102 (13)	0.0522 (14)	0.0107 (15)
C7	0.084 (2)	0.058 (4)	0.062 (3)	0.003 (2)	0.059 (2)	−0.0001 (19)
C6	0.0703 (16)	0.047 (4)	0.066 (2)	−0.001 (3)	0.0567 (16)	−0.002 (2)
C1	0.093 (4)	0.040 (2)	0.132 (5)	0.010 (2)	0.096 (4)	0.012 (3)
C5	0.0517 (13)	0.066 (4)	0.0520 (16)	0.003 (2)	0.0403 (13)	−0.006 (2)
C8	0.123 (4)	0.062 (3)	0.078 (5)	0.003 (3)	0.084 (5)	0.000 (3)
C3A	0.094 (10)	0.25 (3)	0.098 (9)	−0.067 (13)	0.087 (8)	−0.059 (12)
C2	0.163 (6)	0.063 (3)	0.188 (7)	0.031 (3)	0.157 (6)	0.013 (3)
C4	0.176 (8)	0.098 (4)	0.244 (11)	0.024 (5)	0.185 (9)	0.019 (6)
C4A	0.095 (10)	0.087 (9)	0.094 (8)	0.003 (7)	0.050 (8)	−0.011 (7)
C3	0.165 (7)	0.094 (5)	0.291 (13)	0.022 (5)	0.202 (9)	0.007 (6)
C2A	0.095 (8)	0.061 (6)	0.146 (12)	0.022 (5)	0.099 (9)	0.031 (7)
C1A	0.124 (13)	0.057 (9)	0.17 (2)	0.030 (9)	0.127 (16)	0.039 (10)
Sn1A	0.0836 (12)	0.0915 (15)	0.0617 (9)	0.0361 (8)	0.0630 (9)	0.0338 (8)
C5A	0.0517 (13)	0.066 (4)	0.0520 (16)	0.003 (2)	0.0403 (13)	−0.006 (2)
C6A	0.0703 (16)	0.047 (4)	0.066 (2)	−0.001 (3)	0.0567 (16)	−0.002 (2)
C7A	0.084 (2)	0.058 (4)	0.062 (3)	0.003 (2)	0.059 (2)	−0.0001 (19)
C8A	0.123 (4)	0.062 (3)	0.078 (5)	0.003 (3)	0.084 (5)	0.000 (3)

### Geometric parameters (Å, º)

**Table:**

Sn1—O1	2.0041 (17)	C8—H8A	0.9600
Sn1—C17	2.098 (3)	C8—H8B	0.9600
Sn1—C1	2.151 (6)	C8—H8C	0.9600
Sn1—C5	2.110 (5)	C3A—H3AA	0.9700
O1—B1	1.352 (3)	C3A—H3AB	0.9700
O1—Sn1A	2.040 (3)	C3A—C4A	1.546 (13)
O2—H2	0.882 (18)	C3A—C2A	1.439 (12)
O2—B1	1.362 (3)	C2—H2A	0.9700
C18—C17	1.427 (3)	C2—H2B	0.9700
C18—C9	1.439 (3)	C2—C3	1.501 (9)
C18—C13	1.435 (3)	C4—H4A	0.9600
C17—C16	1.380 (3)	C4—H4B	0.9600
C17—Sn1A	2.210 (4)	C4—H4C	0.9600
C9—B1	1.594 (3)	C4—C3	1.489 (8)
C9—C10	1.382 (3)	C4A—H4AA	0.9600
C13—C12	1.401 (4)	C4A—H4AB	0.9600
C13—C14	1.422 (4)	C4A—H4AC	0.9600
C16—H16	0.9300	C3—H3A	0.9700
C16—C15	1.399 (4)	C3—H3B	0.9700
C12—H12	0.9300	C2A—H2AA	0.9700
C12—C11	1.352 (4)	C2A—H2AB	0.9700
C15—H15	0.9300	C2A—C1A	1.483 (13)
C15—C14	1.357 (4)	C1A—H1AA	0.9700
C14—H14	0.9300	C1A—H1AB	0.9700
C10—H10	0.9300	C1A—Sn1A	2.157 (13)
C10—C11	1.410 (3)	Sn1A—C5A	2.154 (12)
C11—H11	0.9300	C5A—H5AA	0.9700
C7—H7A	0.9700	C5A—H5AB	0.9700
C7—H7B	0.9700	C5A—C6A	1.513 (12)
C7—C6	1.500 (6)	C6A—H6AA	0.9700
C7—C8	1.521 (7)	C6A—H6AB	0.9700
C6—H6A	0.9700	C6A—C7A	1.489 (13)
C6—H6B	0.9700	C7A—H7AA	0.9700
C6—C5	1.535 (6)	C7A—H7AB	0.9700
C1—H1A	0.9700	C7A—C8A	1.498 (13)
C1—H1B	0.9700	C8A—H8AA	0.9600
C1—C2	1.530 (9)	C8A—H8AB	0.9600
C5—H5A	0.9700	C8A—H8AC	0.9600
C5—H5B	0.9700		
			
O1—Sn1—C17	99.30 (8)	H8B—C8—H8C	109.5
O1—Sn1—C1	107.0 (3)	H3AA—C3A—H3AB	108.1
O1—Sn1—C5	103.2 (2)	C4A—C3A—H3AA	109.6
C17—Sn1—C1	111.7 (2)	C4A—C3A—H3AB	109.6
C17—Sn1—C5	112.8 (2)	C2A—C3A—H3AA	109.6
C5—Sn1—C1	120.1 (3)	C2A—C3A—H3AB	109.6
B1—O1—Sn1	121.92 (13)	C2A—C3A—C4A	110.4 (11)
B1—O1—Sn1A	124.28 (15)	C1—C2—H2A	108.9
B1—O2—H2	113 (2)	C1—C2—H2B	108.9
C17—C18—C9	123.78 (18)	H2A—C2—H2B	107.7
C17—C18—C13	117.34 (19)	C3—C2—C1	113.2 (7)
C13—C18—C9	118.9 (2)	C3—C2—H2A	108.9
C18—C17—Sn1	121.60 (15)	C3—C2—H2B	108.9
C18—C17—Sn1A	122.42 (16)	H4A—C4—H4B	109.5
C16—C17—Sn1	117.84 (19)	H4A—C4—H4C	109.5
C16—C17—C18	120.5 (2)	H4B—C4—H4C	109.5
C16—C17—Sn1A	117.0 (2)	C3—C4—H4A	109.5
C18—C9—B1	127.12 (19)	C3—C4—H4B	109.5
C10—C9—C18	117.46 (19)	C3—C4—H4C	109.5
C10—C9—B1	115.39 (18)	C3A—C4A—H4AA	109.5
C12—C13—C18	120.0 (2)	C3A—C4A—H4AB	109.5
C12—C13—C14	120.3 (2)	C3A—C4A—H4AC	109.5
C14—C13—C18	119.6 (2)	H4AA—C4A—H4AB	109.5
C17—C16—H16	119.2	H4AA—C4A—H4AC	109.5
C17—C16—C15	121.7 (3)	H4AB—C4A—H4AC	109.5
C15—C16—H16	119.2	C2—C3—H3A	107.0
C13—C12—H12	119.5	C2—C3—H3B	106.9
C11—C12—C13	121.0 (2)	C4—C3—C2	121.5 (9)
C11—C12—H12	119.5	C4—C3—H3A	106.9
C16—C15—H15	120.2	C4—C3—H3B	106.9
C14—C15—C16	119.6 (2)	H3A—C3—H3B	106.7
C14—C15—H15	120.2	C3A—C2A—H2AA	106.9
O1—B1—O2	118.44 (18)	C3A—C2A—H2AB	106.9
O1—B1—C9	126.12 (18)	C3A—C2A—C1A	121.8 (14)
O2—B1—C9	115.44 (18)	H2AA—C2A—H2AB	106.7
C13—C14—H14	119.4	C1A—C2A—H2AA	106.9
C15—C14—C13	121.3 (2)	C1A—C2A—H2AB	106.9
C15—C14—H14	119.4	C2A—C1A—H1AA	108.0
C9—C10—H10	118.5	C2A—C1A—H1AB	108.0
C9—C10—C11	123.1 (2)	C2A—C1A—Sn1A	117.2 (11)
C11—C10—H10	118.5	H1AA—C1A—H1AB	107.2
C12—C11—C10	119.6 (3)	Sn1A—C1A—H1AA	108.0
C12—C11—H11	120.2	Sn1A—C1A—H1AB	108.0
C10—C11—H11	120.2	O1—Sn1A—C17	94.66 (15)
H7A—C7—H7B	107.9	O1—Sn1A—C1A	105.8 (10)
C6—C7—H7A	109.2	O1—Sn1A—C5A	106.7 (7)
C6—C7—H7B	109.2	C1A—Sn1A—C17	110.1 (4)
C6—C7—C8	112.1 (6)	C5A—Sn1A—C17	113.4 (5)
C8—C7—H7A	109.2	C5A—Sn1A—C1A	122.2 (8)
C8—C7—H7B	109.2	Sn1A—C5A—H5AA	108.0
C7—C6—H6A	108.7	Sn1A—C5A—H5AB	108.0
C7—C6—H6B	108.7	H5AA—C5A—H5AB	107.3
C7—C6—C5	114.2 (5)	C6A—C5A—Sn1A	117.2 (11)
H6A—C6—H6B	107.6	C6A—C5A—H5AA	108.0
C5—C6—H6A	108.7	C6A—C5A—H5AB	108.0
C5—C6—H6B	108.7	C5A—C6A—H6AA	108.3
Sn1—C1—H1A	109.1	C5A—C6A—H6AB	108.3
Sn1—C1—H1B	109.1	H6AA—C6A—H6AB	107.4
H1A—C1—H1B	107.8	C7A—C6A—C5A	115.7 (13)
C2—C1—Sn1	112.7 (5)	C7A—C6A—H6AA	108.3
C2—C1—H1A	109.1	C7A—C6A—H6AB	108.3
C2—C1—H1B	109.1	C6A—C7A—H7AA	108.6
Sn1—C5—H5A	109.4	C6A—C7A—H7AB	108.6
Sn1—C5—H5B	109.4	C6A—C7A—C8A	114.7 (14)
C6—C5—Sn1	111.2 (4)	H7AA—C7A—H7AB	107.6
C6—C5—H5A	109.4	C8A—C7A—H7AA	108.6
C6—C5—H5B	109.4	C8A—C7A—H7AB	108.6
H5A—C5—H5B	108.0	C7A—C8A—H8AA	109.5
C7—C8—H8A	109.5	C7A—C8A—H8AB	109.5
C7—C8—H8B	109.5	C7A—C8A—H8AC	109.5
C7—C8—H8C	109.5	H8AA—C8A—H8AB	109.5
H8A—C8—H8B	109.5	H8AA—C8A—H8AC	109.5
H8A—C8—H8C	109.5	H8AB—C8A—H8AC	109.5
			
Sn1—O1—B1—O2	176.60 (15)	C13—C18—C17—C16	−0.2 (3)
Sn1—O1—B1—C9	−4.7 (3)	C13—C18—C17—Sn1A	−176.2 (2)
Sn1—C17—C16—C15	−177.2 (2)	C13—C18—C9—B1	−177.74 (19)
Sn1—C1—C2—C3	59.5 (9)	C13—C18—C9—C10	0.0 (3)
C18—C17—C16—C15	−0.1 (4)	C13—C12—C11—C10	−0.7 (5)
C18—C9—B1—O1	2.6 (3)	C16—C15—C14—C13	−0.6 (4)
C18—C9—B1—O2	−178.7 (2)	C12—C13—C14—C15	179.0 (3)
C18—C9—C10—C11	−1.1 (4)	B1—C9—C10—C11	176.9 (3)
C18—C13—C12—C11	−0.4 (4)	C14—C13—C12—C11	−179.1 (3)
C18—C13—C14—C15	0.3 (4)	C10—C9—B1—O1	−175.2 (2)
C17—C18—C9—B1	1.6 (3)	C10—C9—B1—O2	3.6 (3)
C17—C18—C9—C10	179.3 (2)	C7—C6—C5—Sn1	−177.6 (7)
C17—C18—C13—C12	−178.6 (2)	C1—C2—C3—C4	167.2 (8)
C17—C18—C13—C14	0.1 (3)	C8—C7—C6—C5	−179.4 (8)
C17—C16—C15—C14	0.5 (4)	C3A—C2A—C1A—Sn1A	−66 (3)
C9—C18—C17—Sn1	−2.6 (3)	C4A—C3A—C2A—C1A	−49 (3)
C9—C18—C17—C16	−179.5 (2)	Sn1A—O1—B1—O2	168.6 (2)
C9—C18—C17—Sn1A	4.4 (3)	Sn1A—O1—B1—C9	−12.7 (3)
C9—C18—C13—C12	0.8 (3)	Sn1A—C17—C16—C15	176.1 (3)
C9—C18—C13—C14	179.5 (2)	Sn1A—C5A—C6A—C7A	170.7 (19)
C9—C10—C11—C12	1.5 (5)	C5A—C6A—C7A—C8A	−173 (2)
C13—C18—C17—Sn1	176.78 (15)		

### Hydrogen-bond geometry (Å, º)

**Table:**

*D*—H···*A*	*D*—H	H···*A*	*D*···*A*	*D*—H···*A*
O2—H2···O1^i^	0.88 (2)	1.93 (2)	2.805 (2)	172 (3)

Symmetry code: (i) −*x*+3/2, −*y*+3/2, −*z*.
